# Sleep Quality and Adherence to the Mediterranean Diet in Male Young Adults: An Exploratory Study Among Amateur Soccer Players, Physically Active Individuals, and Individuals with High Sitting Time

**DOI:** 10.3390/sports14090411

**Published:** 2026-09-17

**Authors:** Emanuel Festino, Olga Papale, Francesca Di Rocco, Silvia Zema, Marianna De Maio, Paulo Roberto dos Santos Amorim, Carl Foster, Alessandra Amato, Patrizia Proia, Cristina Cortis, Andrea Fusco

**Affiliations:** 1 Department of Human Sciences, Society and Health, University of Cassino and Lazio Meridionale, 03043 Cassino, Italy; emanuel.festino@unicas.it (E.F.); olga.papale@unicas.it (O.P.); francesca.dirocco@uniroma5.it (F.D.R.); silvia.zema@unicas.it (S.Z.); marianna.demaio@unicas.it (M.D.M.); 2 European University of Technology EUt+, 03043 Cassino, Italy; 3 Department of Human Sciences and Promotion of the Quality of Life, “San Raffaele” Open University of Rome, 00166 Rome, Italy; 4 National Center for Disease Prevention and Health Promotion, Istituto Superiore di Sanità (ISS), 00161 Rome, Italy; 5 Department of Physical Education, Federal University of Viçosa, Viçosa 36570-900, Brazil; pramorim@ufv.br; 6 Department of Exercise and Sport Science, University of Wisconsin-La Crosse, La Crosse, WI 54601, USA; cfosteruwl@gmail.com; 7 Sport and Exercise Sciences Research Unit, Department of Psychology, Educational Science and Human Movement, University of Palermo, 90144 Palermo, Italy; alessandra.amato02@unipa.it (A.A.); patrizia.proia@unipa.it (P.P.); 8 Department of Medicine and Aging Sciences, University “G. d’Annunzio” of Chieti-Pescara, 66100 Chieti, Italy; andrea.fusco@unich.it

**Keywords:** amateur athletes, chronotype, Mediterranean diet adherence, sedentary behavior, sleep disturbance

## Abstract

Amateur soccer players represent a relatively understudied population and often combine training with study or work, facing daily challenges that can impact sleep, diet, and circadian-related behaviors. Since these factors are important for health, recovery, and well-being, this study aimed to examine sleep quality, adherence to the Mediterranean diet, and chronotype in amateur soccer players compared with physically active individuals and individuals with high sitting time. The sample comprised 55 male young adults (23.36 ± 3.11 years): amateur soccer players (*n* = 20), physically active individuals (*n* = 18), and individuals with high sitting time (*n* = 17). All participants completed the Pittsburgh Sleep Quality Index (PSQI), the PREvención con DIeta MEDiterránea (PREDIMED), and the Morningness–Eveningness Questionnaire (MEQ). Group comparisons were performed using appropriate parametric or non-parametric tests, and exploratory correlation analyses were used to examine associations among sleep quality, Mediterranean diet adherence, and chronotype. Lower PSQI scores (*p* < 0.001) were observed in soccer players (3.80 ± 1.32) and physically active individuals (4.17 ± 1.92) than in individuals with high sitting time (6.24 ± 2.05). No significant between-group differences were found in PREDIMED (soccer players = 6.80 ± 2.22; physically active individuals = 7.50 ± 1.89; individuals with high sitting time = 7.29 ± 2.42) and MEQ (soccer players = 51.60 ± 5.90; physically active individuals = 50.89 ± 7.70; individuals with high sitting time = 51.82 ± 11.43). Exploratory correlation analyses, without correction for multiple testing, identified a negative association between MEQ and PSQI in individuals with high sitting time (*r* = −0.514, *p* = 0.035). These findings describe group differences in self-reported sleep quality, whereas Mediterranean diet adherence and chronotype appeared similar across groups.

## 1. Introduction

Sleep plays a key role in health and well-being [1]. Poor sleep quality has been linked to mental health issues and reduced overall well-being and is recognized as an important indicator of cardiovascular health [1,2,3]. This is particularly relevant in young adults, who often experience irregular schedules due to academic, occupational, and social demands. Given its impact on both physical and psychological functioning, sleep quality represents a central outcome in lifestyle and health research.

Sleep quality is associated with multiple behavioral and biological factors. Among lifestyle-related factors, adherence to the Mediterranean diet has been associated with sleep duration and indicators of sleep quality [4]. Although these associations do not imply causality, they suggest that dietary patterns should be considered when examining sleep-related outcomes. Chronotype, which reflects individual preferences for the timing of sleep–wake behavior and daily activities, may also be related to sleep quality [5,6]. Circadian preference, particularly eveningness, and misalignment between preferred biological timing and externally imposed schedules have been associated with sleep-related outcomes [5,6]. Thus, dietary adherence and chronotype represent complementary characteristics that can be examined alongside sleep quality without implying causal relationships among these factors.

Physical exercise is generally associated with better sleep quality [7]. However, the relationship between physical exercise and sleep may vary according to training volume, intensity, timing, and the adequacy of recovery [7,8,9]. Poorly managed training loads combined with insufficient recovery have been associated with fatigue and sleep disturbances, while inadequate sleep may, in turn, compromise recovery and adaptation to training [7,9,10]. If sustained, this imbalance may negatively affect well-being and contribute to maladaptive responses to training [7,10]. In the sports context, recovery is a key determinant of health and performance, particularly in disciplines characterized by high training loads, such as soccer [11,12]. Sleep is a key component of recovery, health, and performance, while high training loads, travel demands, and performance-related pressure have been associated with poor sleep quality [13,14,15,16]. Training timing represents an additional factor that may be relevant to athletes’ sleep. In fact, athletes training late in the evening may experience greater sleep difficulties than those training during daytime hours [17]. However, the relationship between evening exercise and sleep may depend on chronotype and habitual training schedules. Previous evidence [18] suggests that the effects of afternoon versus early-evening high-intensity exercise on objective sleep may differ according to athletes’ chronotype, whereas late-evening exercise did not significantly alter objective or subjective sleep in soccer players accustomed to training late in the evening [19]. These findings [18,19] suggest that both exercise timing and its alignment with individual circadian preference and habitual routines should be considered when examining sleep in athletic populations.

Most existing research has focused on elite or professional athletic populations, including professional soccer players and elite adolescent athletes from other sports [15,20,21], who typically train in highly structured settings, receive nutritional and medical support, and rarely need to balance sport participation with occupational demands. However, amateur soccer players represent a substantial proportion of the sporting population in Italy, given the widespread participation in soccer, particularly at the amateur level [22], and often combine training and competition with work or university commitments. Within this context, achieving a satisfactory work–life balance is particularly challenging [23,24], as these commitments can reduce the time available for training and recovery [25,26]. Training sessions are often scheduled in the evening, close to bedtime, which may influence sleep–wake regulation and sleep outcomes [25,27]. Recent evidence indicates that sleep-related issues are also relevant outside elite sport [28,29]. In a large sample of Italian athletes, sleep characteristics differed according to competitive level, with non-elite athletes showing lower sleep efficiency and greater sleep latency and wake after sleep onset than elite athletes [28]. Sleep quality and chronotype have also been investigated in young male players from a non-professional soccer team, further supporting the relevance of these factors in non-professional soccer settings [29]. Moreover, studies on dual-career athletes [14,30,31] indicate that the combined demands of education and training are associated with variations in sleep quality, with academic workload, study time, and training demands emerging as relevant factors. This combination of occupational, academic, and sport-related demands makes amateur soccer players an important but understudied population in sleep and behavioral health research. Recent research has also applied a multidimensional approach incorporating sleep quality, Mediterranean diet adherence, chronotype, and physical activity [32]. Within this framework, sleep quality, dietary adherence, and chronotype can be considered complementary aspects of daily functioning [14,32], particularly relevant in populations with heterogeneous routines, such as amateur athletes and young adults. However, studying amateur soccer players alone cannot determine whether observed differences are specific to soccer participation or are also present in individuals engaged in regular structured physical activity. Including a physically active comparison group may help distinguish soccer-specific patterns from those associated more broadly with regular exercise, while a high-sitting-time group provides a complementary behavioral contrast based on prolonged sedentary exposure. Because these groups may also differ in training routines, sitting behavior, and other lifestyle characteristics, comparisons should be interpreted as differences between habitual sport and activity profiles rather than as evidence of a causal effect of physical activity. Accordingly, it remains unclear how sleep quality, Mediterranean diet adherence, and chronotype differ across amateur soccer players, physically active individuals, and individuals with high sitting time. Therefore, the primary aim of this study was to compare these characteristics across the three groups. A secondary aim was to explore the associations of sleep quality with Mediterranean diet adherence and chronotype within each group. Based on previous evidence [14,18,33] reporting associations between activity-related behaviors, sedentary behavior, and sleep quality, we hypothesized that PSQI scores would differ among groups.

## 2. Materials and Methods

### 2.1. Participants

This cross-sectional study was conducted in accordance with the Declaration of Helsinki and was approved by the Institutional Review Board of the Department of Human Sciences, Society and Health of the University of Cassino and Lazio Meridionale (8 March 2023, approval number 9407). All participants provided written informed consent and were informed that they could withdraw from the study at any time without providing a reason. Because the study was exploratory, no a priori sample-size calculation was performed. Recruitment and data collection took place between April and May 2023. Participation was voluntary, and no financial or material incentives were provided. Participants were recruited using a non-probability convenience sampling approach from two sources: a regional-level amateur soccer team and the university population. No random sampling procedure was applied. Predefined eligibility and group-classification criteria were subsequently used to define the three study groups.

Twenty male soccer players were recruited from a regional-level amateur soccer team officially affiliated with the Italian Football Federation. All players regularly participated in structured team training sessions three times per week, each lasting approximately 90 min, in addition to one official match per week. Training was conducted in a team setting and included the usual components of amateur soccer practice, such as technical–tactical drills and physical conditioning. Training sessions were typically scheduled in the evening, starting at 19:00. Because soccer players were recruited from the same team, they shared a similar sport-specific context in terms of training schedule, competitive level, and environmental conditions. Participants were required to have a minimum of four years of continuous soccer practice at the amateur level and to be actively engaged in both training and competition at the time of the study. Players were excluded if they reported any injury or medical condition that prevented full participation in training sessions or official matches. These criteria were adopted to ensure a stable training background and increase sample homogeneity. Recruitment and data collection for soccer players occurred during the competitive season. Training sessions were conducted on weekdays, whereas official matches were typically played on weekends, and travel-related demands were not a factor during the weekdays.

To allow for peer comparisons, eighteen physically active individuals and seventeen individuals with high sitting time were recruited from the university population. Physical activity levels and sedentary behavior were assessed using the Italian short version (7 items) of the International Physical Activity Questionnaire (IPAQ) [34]. Physically active individuals were classified as health-enhancing physical activity (HEPA) active according to established IPAQ criteria [35]. All participants in this group reported engaging in structured fitness-based physical activity three times per week, with each session lasting approximately 90 min. Activities were performed in university gym facilities and generally included resistance-training exercises targeting the major muscle groups, using both free weights and standard resistance machines, together with functional exercises involving body-weight movements and commonly available gym equipment. Sessions were organized in a structured fitness format and were primarily aimed at strength and hypertrophy development. These activities reflected the participants’ habitual training routines and were not prescribed or standardized by the research team. Training sessions were typically scheduled in the late afternoon, after university lectures, at approximately 18:00. Individuals with high sitting time were identified based on the IPAQ sitting item, which asks participants to report the time spent sitting on a weekday during the previous seven days, including time spent at work, at home, during coursework, and leisure activities [35]. Participants reporting five or more hours of sitting per day were included in this group. This criterion was selected because prolonged sedentary behavior is recognized as an independent risk factor for adverse health outcomes, including cardiometabolic disorders and psychological distress [36,37,38,39,40,41]. Classification was based on sedentary exposure rather than physical inactivity and was independent of the IPAQ physical activity category, as individuals who meet recommended levels of physical activity may still accumulate substantial sitting time [14,40,42]. This approach allowed participants selected for regular structured physical activity to be compared with those characterized by prolonged sitting without assuming physical inactivity in the latter group. Amateur soccer players were recruited separately as a sport-specific group based on regular participation in training and competition. Sitting time was not assessed in this group; therefore, soccer players were not classified according to the criterion used for the high-sitting-time group.

### 2.2. Procedures

**Figure 1:**
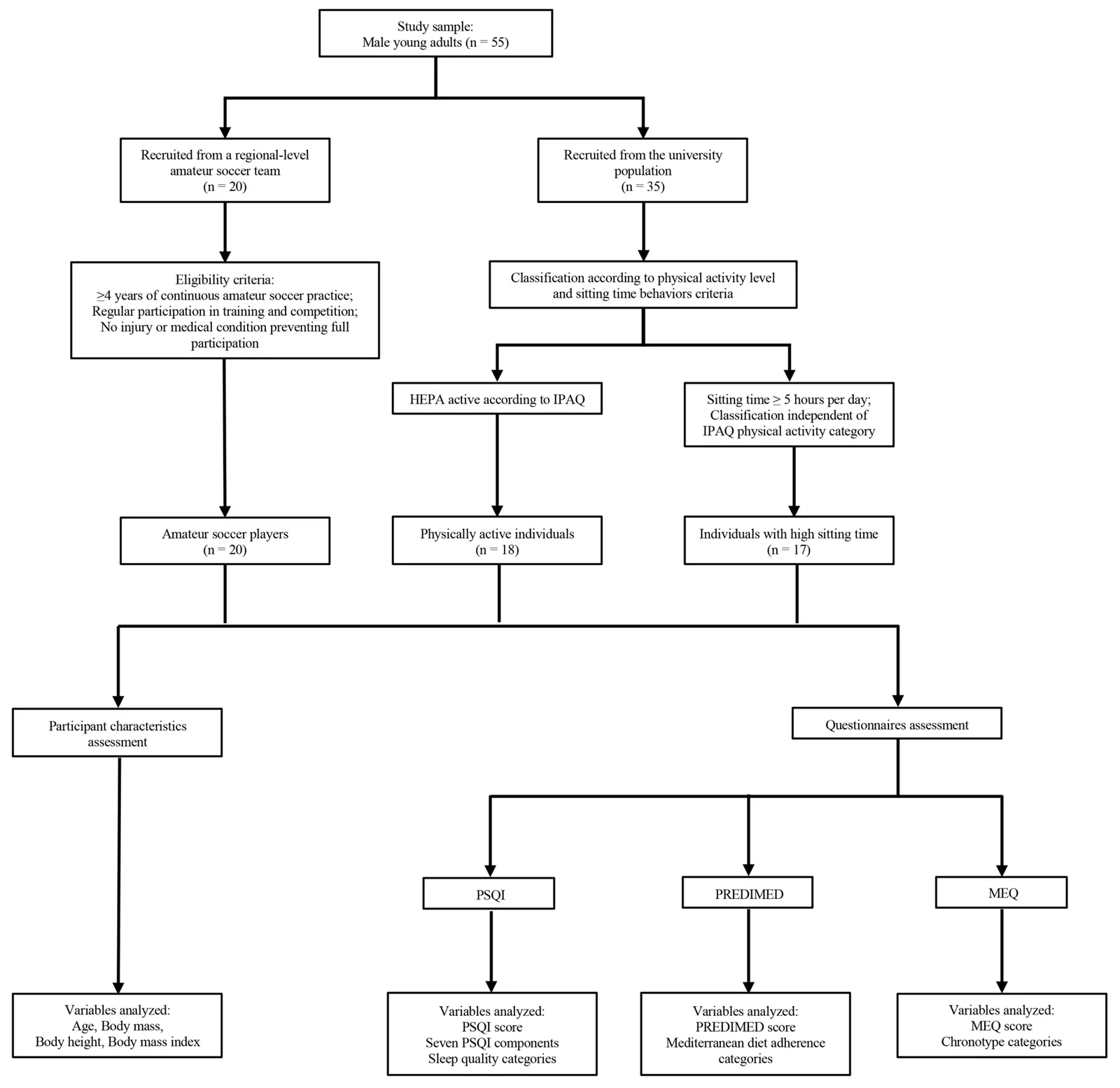
Flowchart of participant recruitment, eligibility and group-classification procedures, assessment procedures, and variables analyzed. HEPA = health-enhancing physical activity; IPAQ = International Physical Activity Questionnaire; PSQI = Pittsburgh Sleep Quality Index; PREDIMED = PREvención con DIeta MEDiterránea; MEQ = Morningness–Eveningness Questionnaire.

Body weight (kg) and height (m) were measured using a Seca 709 scale with an integrated stadiometer (Vogel & Halke, Hamburg, Germany), with a precision of 0.1 kg and 0.1 cm, respectively. Body mass index (BMI) was calculated as weight in kilograms divided by the square of height in meters (kg/m^2^). During the session, participants were asked to complete a total of three questionnaires (fully described in subsequent sections): the Pittsburgh Sleep Quality Index (PSQI), the questionnaire for the assessment of PREvención con DIeta MEDiterránea (PREDIMED), and the Morningness–Eveningness Questionnaire (MEQ). All questionnaires were administered in a quiet environment in the Human Performance Lab of the University of Cassino and Lazio Meridionale to reduce distraction and improve response accuracy. The total time to complete the questionnaires was approximately 20 min. Soccer players and physically active individuals completed the questionnaires during laboratory visits conducted before any exercise on the day of assessment, whereas individuals with high sitting time completed them during a comparable weekday laboratory visit. All assessments were conducted prior to exercise rather than immediately following an exercise session to avoid acute exercise-related effects on questionnaire responses. Participant recruitment, eligibility, and group-classification procedures, assessments, and variables analyzed are summarized in Figure 1.

#### 2.2.1. Pittsburgh Sleep Quality Index

The Italian version of the PSQI was used to evaluate subjective sleep quality and disturbances over the previous month. It includes 19 questions grouped into seven components: subjective sleep quality, sleep latency, sleep duration, habitual sleep efficiency, sleep disturbance, use of sleeping medications, and daytime dysfunction. Each component is scored on a scale from 0 to 3, with higher scores indicating greater dysfunction. The sum of the component scores provides a total score ranging from 0 to 21, with 0 indicating no sleep issues and 21 indicating severe sleep difficulties and low sleep quality. A PSQI score below 5 indicates good sleep quality, whereas scores ≥ 5 denote poor sleep quality [43].

#### 2.2.2. Questionnaire for the Assessment of PREvención Con DIeta MEDiterránea

The Italian version of the 14-item PREDIMED questionnaire was used to assess adherence to the Mediterranean diet. The PREDIMED questionnaire consists of 14 items assessing food-consumption frequency and dietary habits related to extra-virgin olive oil, fruit, vegetables, nuts, legumes, red meat, poultry, fish, animal fat, sweetened beverages, sweets, and dishes seasoned with sofrito. Responses consistent with the Mediterranean dietary pattern were scored 1; all other responses were scored 0. The total PREDIMED score was calculated by summing the scores across the 14 items. Based on established cutoffs, adherence was categorized as follows: a score ≤ 5 indicated the lowest adherence, scores between 6 and 9 indicated average adherence, and a score ≥ 10 indicated the highest adherence [44]. Although the PREDIMED questionnaire was validated in middle-aged and older adults at high cardiovascular risk, it has been applied in Italian samples across adult populations, including young adults [14,45,46]. However, a validation specifically targeting young adults or athletic populations is currently lacking. For this reason, the questionnaire was used to provide a brief estimate of adherence to a dietary pattern rather than a detailed assessment of nutrient intake or sport-specific nutritional practices. The questionnaire was self-administered by all participants. To facilitate understanding, participants were provided with standardized explanations of culturally specific food items (e.g., portion sizes or traditional dishes) by a member of the research team before completing the questionnaire.

#### 2.2.3. Morningness–Eveningness Questionnaire

The Italian version of the MEQ was used to assess individual preferences for the timing of daily activities (i.e., chronotype). The questionnaire includes both Likert-type and time-based items. Likert-type items offer four options, with lower scores indicating a stronger evening preference. Time-based items are scored based on selected time intervals over a 7-h range, with all responses scored from 1 to 5. The total score is the sum of all item scores, and it is used to classify chronotype into five categories: definitely morning type (70–86), moderately morning type (59–69), neither type (42–58), moderately evening type (31–41), and definitely evening type (16–30) [47].

### 2.3. Statistical Analysis

Continuous variables are expressed as mean, standard deviation, and median, whereas categorical variables are reported as frequencies and percentages (%). The continuous variables were age, body mass, body height, BMI, PSQI score, PREDIMED score, and MEQ score. The seven PSQI components were treated as ordinal variables. The categorical variables were good sleep quality and poor sleep quality for PSQI, while low adherence, average adherence, and high adherence for PREDIMED. For MEQ, the categorical variables were definitely morning, moderately morning, neither, moderately evening, and definitely evening. The Shapiro–Wilk test was used to assess the normal distribution of the data.

Participants’ characteristics (age, body mass, body height, and BMI) were compared among the three groups (soccer players, physically active individuals, and individuals with high sitting time). Variables showing departures from normality in at least one group were analyzed using the Kruskal–Wallis test, whereas one-way Analysis of Variance (ANOVA) was used for normally distributed variables. One-way ANOVA was used to compare sleep quality (PSQI score), adherence to the Mediterranean diet (PREDIMED score), and chronotype (MEQ score) among the three groups. Homogeneity of variances was assessed using Levene’s test. When this assumption was violated, Welch’s ANOVA was applied. When a significant overall group effect was detected, Bonferroni-adjusted post hoc comparisons were performed. Because the seven PSQI components were not normally distributed, between-group differences were assessed using Kruskal–Wallis tests. When significant main effects were found, pairwise post hoc comparisons were performed using Dunn’s test with Bonferroni adjustment. Effect sizes were calculated and interpreted using Cohen’s *d* and eta squared (η^2^) for parametric analyses [48], while Rosenthal’s *r* was used for non-parametric tests [49]. Differences in the distribution of sleep quality categories (good vs. poor sleep quality), Mediterranean diet adherence categories (low, average, and high), and chronotype categories (definitely morning, moderately morning, neither, moderately evening, and definitely evening) were assessed using Pearson’s chi-square (χ^2^) test. When expected cell counts were below 5, the Fisher–Freeman–Halton exact test with Monte Carlo estimation based on 10,000 sampled tables was used.

The Firth penalized logistic regression model was performed as an exploratory and sensitivity analysis to examine the association between study group and poor sleep quality. The Firth penalized logistic regression was used to reduce small-sample bias. Poor sleep quality was coded as a binary outcome according to the PSQI cutoff. Soccer players were used as the reference group, and two indicator variables were included to compare physically active individuals and individuals with high sitting time with amateur soccer players. PREDIMED and MEQ scores were included as continuous covariates. Odds ratios (ORs) with 95% profile-penalized likelihood confidence intervals (CIs) were reported. Penalized likelihood-ratio tests were used to assess the statistical significance of the overall model and the joint group term (physically active individuals vs. amateur soccer players; individuals with high sitting time vs. amateur soccer players). Multicollinearity among the predictors was assessed using tolerance and variance inflation factor values. Given the exploratory design and small sample size, the regression analysis was not intended to provide a fully confounder-adjusted estimate of the independent association of physical activity or high sitting time with sleep quality. Potential confounding factors that were not collected as standardized participant-level variables could not be incorporated into the model. Therefore, the regression estimates were interpreted as exploratory associations rather than fully adjusted estimates.

Exploratory Pearson’s correlation coefficients (*r*) were calculated to examine the associations between sleep quality (PSQI score), adherence to the Mediterranean diet (PREDIMED score), and chronotype (MEQ score). Correlation analyses were conducted separately for soccer players, physically active individuals, and individuals with high sitting time. All statistical tests were two-tailed, with a *p*-value (*p*) < 0.05 considered statistically significant. Where appropriate, 95% CIs were reported for group mean differences and correlation coefficients, and SEs were reported for pairwise group comparisons. The statistical analysis was performed using IBM SPSS Statistics, Version 31.0 (IBM Corp, Armonk, NY, USA), except for the Firth penalized logistic regression, which was performed using R version 4.4.1 (R Foundation for Statistical Computing, Vienna, Austria) through the R integration environment in SPSS, using the logistf package (version 1.26.1).

## 3. Results

The characteristics of the participants are reported in Table 1.

Shapiro–Wilk tests indicated that the participant characteristics significantly differed from a normal distribution in soccer players (age: *p* = 0.014; body mass: *p* = 0.001; BMI: *p* = 0.001) and individuals with high sitting time (age: *p* = 0.025; BMI: *p* = 0.001), whereas no significant deviations from normality were observed in physically active individuals (age: *p* = 0.127; BMI: *p* = 0.480). Moreover, body mass was normally distributed in physically active individuals (*p* = 0.436) and in individuals with high sitting time (*p* = 0.086). Body height did not significantly differ from a normal distribution in soccer players (*p* = 0.144), physically active individuals (*p* = 0.287), or individuals with high sitting time (*p* = 0.344). The PSQI score did not significantly deviate from normality in soccer players (*p* = 0.085), physically active individuals (*p* = 0.052), and individuals with high sitting time (*p* = 0.477). The seven PSQI components were not normally distributed across the three groups: subjective sleep quality (all *p* < 0.001), sleep latency (all *p* ≤ 0.006), sleep duration (all *p* ≤ 0.003), habitual sleep efficiency (all *p* < 0.001), sleep disturbances (all *p* < 0.001), use of sleeping medications (all *p* < 0.001), and daytime dysfunction (all *p* < 0.001). PREDIMED score and MEQ score were normally distributed in soccer players (PREDIMED score: *p* = 0.251; MEQ score: *p* = 0.795), physically active individuals (PREDIMED score: *p* = 0.717; MEQ score: *p* = 0.252), and individuals with high sitting time (PREDIMED score: *p* = 0.306; MEQ score: *p* = 0.291). Levene’s test indicated homogeneity of variances for the PSQI score (*p* = 0.328) and PREDIMED score (*p* = 0.644), whereas the assumption was violated for body height (*p* = 0.029) and MEQ (*p* = 0.018). Therefore, Welch’s ANOVA was used to compare body height and MEQ scores between groups. Because several expected cell counts were below 5, group differences were assessed using the Fisher–Freeman–Halton exact test with Monte Carlo estimation for both Mediterranean diet adherence and chronotype categories.

Kruskal–Wallis tests showed no significant group differences for age (χ^2^_(2)_ = 1.78, *p* = 0.410), body mass (χ^2^_(2)_ = 0.55, *p* = 0.759), or BMI (χ^2^_(2)_ = 3.91, *p* = 0.141). Moreover, Welch’s ANOVA revealed no significant group effect for body height (Welch’s F_(2, 31.98)_ = 1.73, *p* = 0.194).

Means and standard deviations for the PSQI score, PREDIMED score, and MEQ score across groups are shown in Table 2.

**Figure 2:**
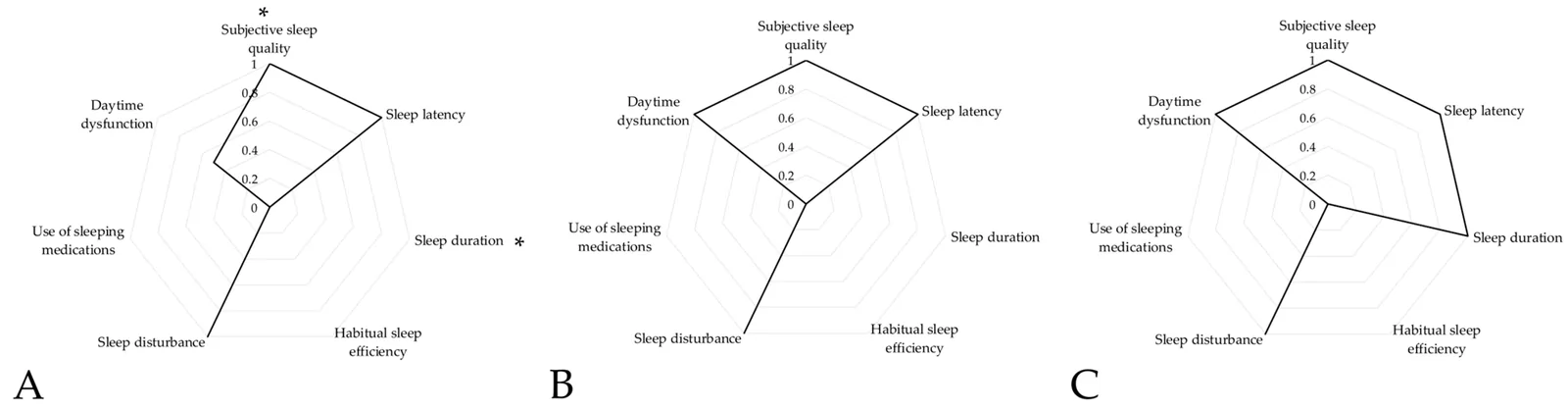
Radar chart showing the median scores of the seven Pittsburgh Sleep Quality Index components across groups: (**A**) amateur soccer players, (**B**) physically active individuals, and (**C**) individuals with high sitting time; * indicates components for which amateur soccer players differed significantly (*p*-value < 0.05) from individuals with high sitting time.

One-way ANOVA revealed a significant between-group difference in PSQI score (F_(2,52)_ = 9.80, *p* < 0.001, η^2^ = 0.27). Post hoc Bonferroni comparisons indicated that individuals with high sitting time reported a higher PSQI score than soccer players (*p* < 0.001, 95% CI = 0.99 to 3.88, SE = 0.58, *d* = 1.44) and physically active individuals (*p* = 0.003, 95% CI = 0.59 to 3.55, SE = 0.60, *d* = 1.04). For the sleep quality components, Kruskal–Wallis tests showed significant group differences for sleep duration (χ^2^_(2)_ = 8.53, *p* = 0.014) and subjective sleep quality (χ^2^_(2)_ = 8.44, *p* = 0.015). Pairwise comparisons indicated that individuals with high sitting time reported shorter sleep duration (*p* = 0.016, SE = 4.44, *r* = 0.46) and poorer subjective sleep quality (*p* = 0.012, SE = 4.81, *r* = 0.48) compared to soccer players. No significant differences were observed between individuals with high sitting time and physically active individuals for any of the PSQI components. Figure 2 shows the graphical representation of the seven PSQI components across groups.

Pearson’s χ^2^ test showed a significant difference in PSQI categories (χ^2^_(2)_ = 15.03, *p* < 0.001). Good sleep quality was more frequent among soccer players (75.00%) and physically active individuals (72.20%) than among individuals with high sitting time (17.60%).

Regarding PREDIMED (F_(2,52)_ = 0.53, *p* = 0.597, η^2^ = 0.02), no significant group effect was observed. For PREDIMED categories, no significant differences were observed between soccer players, physically active individuals, and individuals with high sitting time (Monte Carlo *p* = 0.932). Most participants were classified within the average adherence category (soccer players = 60.00%; physically active individuals = 72.20%; individuals with high sitting time = 58.80%), while only a small percentage was classified as high adherence (soccer players = 15.00%; physically active individuals = 11.10%; individuals with high sitting time = 17.60%).

Welch’s ANOVA revealed no significant group effect for MEQ (F_(2, 31.05)_ = 0.061, *p* = 0.941, η^2^ = 0.002). For MEQ categories, no significant differences were observed between soccer players, physically active individuals, and individuals with high sitting time (Monte Carlo *p* = 0.136). Most participants were identified as neither chronotype (soccer players = 85.00%; physically active individuals = 77.80%; individuals with high sitting time = 47.10%). Few participants were classified as moderately morning (soccer players = 10.00%; physically active individuals = 16.70%; individuals with high sitting time = 17.60%) and moderately evening chronotype (soccer players = 5.00%; physically active individuals = 5.60%; individuals with high sitting time = 23.50%).

In the Firth penalized logistic regression model, the overall model was statistically significant (penalized likelihood-ratio test = 16.30, *p* = 0.003), and the joint group term was also statistically significant (penalized likelihood-ratio test = 15.17, *p* < 0.001). Individuals with high sitting time showed higher odds of poor sleep quality compared with soccer players (OR = 12.71, 95% profile penalized likelihood CI = 2.91 to 76.77, *p* < 0.001). Physically active individuals did not differ from soccer players in the odds of poor sleep quality (OR = 1.13, 95% profile penalized likelihood CI = 0.27 to 4.74, *p* = 0.861). PREDIMED score was not significantly associated with poor sleep quality (OR = 0.94, 95% profile penalized likelihood CI = 0.69 to 1.26, *p* = 0.680), nor was MEQ score (OR = 0.95, 95% profile penalized likelihood CI = 0.87 to 1.03, *p* = 0.198). Multicollinearity was not observed, with tolerance values ranging from 0.77 to 0.96 and variance inflation factor values ranging from 1.05 to 1.31.

**Figure 3:**
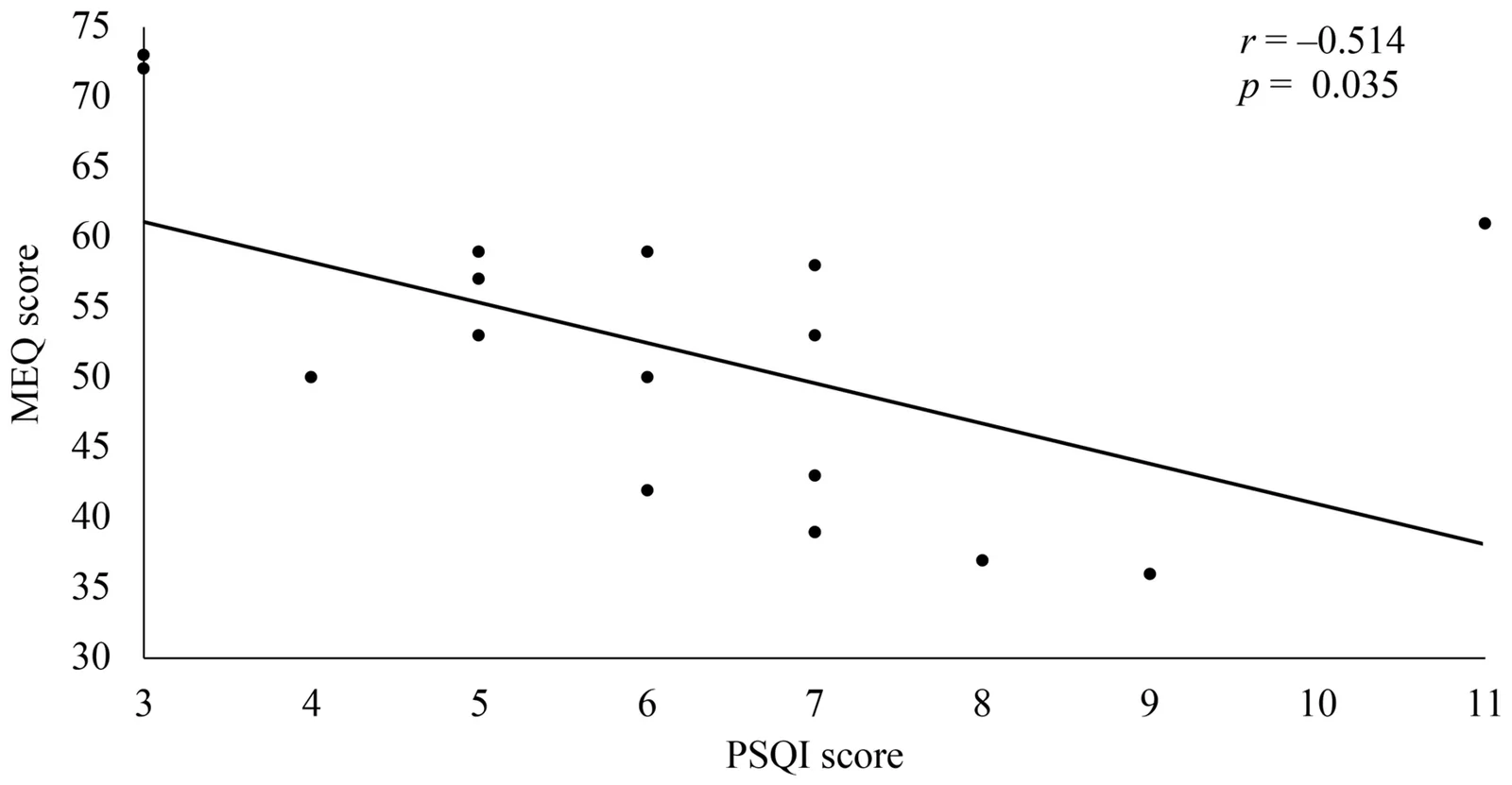
Scatter plot showing the relationship between Pittsburgh Sleep Quality Index (PSQI) score and Morningness–Eveningness Questionnaire (MEQ) score in individuals with high sitting time. Pearson’s correlation coefficient (*r*) and corresponding *p*-value (*p*) are displayed in the figure.

No significant correlations (Table 3) were observed between PSQI and PREDIMED scores in any group or between PSQI and MEQ scores in soccer players and physically active individuals. Across all correlation analyses, only one association reached statistical significance. Specifically, a negative correlation (Figure 3) was found between PSQI score and MEQ score in individuals with high sitting time, indicating that poorer sleep quality was associated with a greater evening preference in individuals with high sitting time. No correction for multiple testing was applied to these exploratory analyses.

## 4. Discussion

This study investigated sleep quality, adherence to the Mediterranean diet, and chronotype in amateur soccer players compared with physically active individuals and individuals with high sitting time. Overall, the findings suggest that sleep quality differed across groups, whereas adherence to the Mediterranean diet and chronotype were similar.

The main finding of the present study was the between-group difference in sleep quality. Individuals with high sitting time reported higher PSQI scores than both amateur soccer players and physically active individuals, with large effect sizes. This finding indicates that high sitting time was associated with poorer sleep quality. However, the cross-sectional design does not allow the direction of this association to be established. Greater sitting time may be associated with poorer sleep, but poorer sleepers may also be more likely to adopt routines characterized by prolonged sitting, while shared lifestyle or behavioral factors may contribute to both. This pattern was further supported by the exploratory Firth penalized logistic regression, which showed higher odds of poor sleep quality in individuals with high sitting time compared with amateur soccer players. However, despite the statistical significance of this association, the wide 95% profile penalized likelihood CI indicates substantial imprecision in its magnitude. Therefore, the estimated OR should be interpreted as exploratory evidence rather than as a precise estimate of the magnitude of the association. In contrast, physically active individuals did not differ from soccer players, indicating that the main contrast in sleep quality was between individuals with high sitting time and the other two groups. The categorical distribution of PSQI scores further highlights the practical relevance of this finding. Approximately three-quarters of soccer players and physically active individuals were classified as good sleepers, whereas fewer than one in five individuals with high sitting time met this criterion. This difference indicates a greater prevalence of self-reported sleep difficulties in individuals with high sitting time than in amateur soccer players or physically active individuals.

The good sleep quality observed in the present soccer players contrasts with previous reports of sleep difficulties in elite or professional athletes [50]. Sleep disruption in competitive sports may be related to high training volumes, irregular schedules, pre-competition anxiety, travel demands, and early-morning training [14,50,51]. Professional athletes may be exposed to greater performance-related pressure because competitive outcomes can directly affect selection, career progression, contractual status, and professional expectations [15,50]. Amateur athletes, although still exposed to competitive stress, may experience a different psychological and recovery context. These differences could partly contribute to the relatively good sleep quality observed in the present amateur soccer players compared with that reported in professional athletes [50]. However, performance pressure and related factors were not directly assessed, and this should be interpreted with caution as descriptive observations. Moreover, the similar sleep quality observed in amateur soccer players and physically active individuals suggests that the observed pattern was not specific to amateur soccer participation. Rather, better sleep quality was observed in both groups characterized by regular sport or structured physical activity. This pattern suggests that the observed differences may be related to the broader contrast between regular engagement in sport or structured physical activity and a lifestyle characterized by prolonged sitting time. However, the present design does not allow for determining whether these differences are primarily related to structured physical activity, sitting time behavior, or other lifestyle and contextual characteristics that differed between groups. The analysis of the seven PSQI components provided a descriptive indication of which sleep dimensions were most involved in the observed group differences in PSQI scores. Specifically, soccer players differed from individuals with high sitting time mainly in sleep duration and subjective sleep quality. Therefore, the lower PSQI total scores observed in soccer players were primarily reflected in lower scores for these two components. No individual PSQI component differed significantly between physically active individuals and those with high sitting time, suggesting that the difference in PSQI scores reflected the combined contribution of multiple sleep dimensions rather than a single component.

No significant between-group difference was observed in Mediterranean diet adherence. Although previous studies [14,52,53] have examined Mediterranean diet adherence and its relationship with performance- and recovery-related outcomes in athletic populations, our results suggest that amateur soccer players do not exhibit particularly high adherence to this dietary pattern. Amateur athletes may have less access to structured nutritional support than professional athletes, where nutrition and recovery strategies are commonly integrated into athlete support [54,55]. This difference could contribute to the average adherence observed in the present soccer players. However, nutritional support and dietary guidance were not assessed, and this interpretation remains speculative. In addition, all participants lived in the same sociocultural context, which may have contributed to similar Mediterranean diet adherence across groups. As a result, most participants reported average adherence, with a relatively small number of individuals showing high adherence to the Mediterranean diet. These findings could suggest that sport participation alone was not associated with greater adherence in the present sample, highlighting the need for further research on Mediterranean dietary patterns and nutritional support in amateur athletic settings [56,57]. No significant between-group differences were observed for chronotype, and most participants were classified as neither type. However, this does not exclude a potential role of training timing in sleep quality. Soccer players typically trained at approximately 19:00, whereas physically active individuals generally trained around 18:00. These times represented typical group schedules rather than individualized exposure measures, and actual sleep timing, the interval between training and bedtime, and chronotype-schedule alignment were not assessed. Therefore, the potential contribution of training timing to the observed sleep patterns cannot be determined. The lower PSQI scores observed in soccer players and physically active individuals than in individuals with high sitting time do not appear to be explained by differences in chronotype alone. Instead, this pattern may reflect differences in sport participation, structured physical activity, sitting time behavior, daily routines, and other lifestyle characteristics across groups.

Correlation analyses provided additional exploratory insights. No significant associations were observed between PSQI and PREDIMED scores in any group. However, most participants were classified within the average adherence category, and this restricted variability, together with the limited subgroup sample sizes, may have reduced the ability to identify associations. These findings should therefore be interpreted as an absence of associations within this sample rather than as evidence that Mediterranean diet adherence and sleep quality are unrelated. Although chronotype did not differ between groups, a negative correlation was observed between PSQI and MEQ scores in individuals with high sitting time, suggesting that poorer sleep quality tended to co-occur with greater evening preference. Epidemiological studies [58,59,60,61] have linked a circadian preference for eveningness with multiple health risks, including sleep problems, poor mental health, and reduced life expectancy. Evening-type individuals may experience shorter sleep duration due to delayed sleep onset and fixed morning activities, further promoting circadian misalignment [33,62]. However, chronotype reflects circadian preference rather than actual sleep behavior, and the mechanism underlying the association observed cannot be determined. Given the exploratory within-group analyses and the absence of correction for multiple testing, this isolated association should be considered hypothesis-generating.

Despite these findings, the study presents several limitations. First, no a priori sample-size calculation was performed because the study was designed as exploratory. The sample size may therefore have limited the ability to identify smaller between-group differences, particularly for PREDIMED and MEQ scores, and reduced the precision of the within-group correlation analyses. Generalizability is also limited by the inclusion of only male young adults and by the recruitment of all amateur soccer players from a single regional-level team. Accordingly, the findings may not be generalizable to female athletes, soccer players from other clubs or competitive levels, or populations with different demographic and sporting characteristics. Second, the study relied on self-reported measures of sleep quality, dietary adherence, chronotype, physical activity, and sitting time. These measures may have introduced subjective bias and did not allow objective evaluation of sleep, physical activity, sitting behavior, or dietary intake. Therefore, in the present context, PREDIMED should be interpreted as a brief measure of Mediterranean diet adherence rather than a detailed assessment of dietary intake. Third, individuals with high sitting time were identified according to self-reported sitting time, independently of their IPAQ physical activity category, whereas amateur soccer players were recruited according to their participation in structured soccer training and competition and were not assessed for sitting time. Consequently, it cannot be determined whether some soccer players also accumulated high levels of sitting time, and the groups should not be interpreted as exclusive categories of physical activity behavior. Fourth, the cross-sectional design prevents causal or directional inference and does not allow the independent contributions of structured physical activity, sitting behavior, and other contextual characteristics to the observed sleep differences to be isolated. Lastly, potential confounding variables such as stress, caffeine and alcohol intake, alignment between chronotype and training time, detailed academic or occupational schedules, post-training nutritional and social habits, and electronic device use before bedtime, which could have influenced sleep and chronotype outcomes, were not controlled.

The present exploratory study focused on selected behavioral and circadian dimensions and was not designed to provide a comprehensive characterization of the broader social, behavioral, and environmental context that may contribute to sleep-related outcomes. Therefore, future studies should address these limitations by including larger and more diverse samples, adopting longitudinal designs, and incorporating objective assessment methods. Physical activity and sitting behavior should be quantified using accelerometry, while sleep should be assessed using actigraphy or, where appropriate, polysomnography. More detailed dietary assessment and repeated measurements of training timing and sleep schedules would provide a more comprehensive characterization of participants’ behavioral profiles. Larger samples would also permit multivariable models capable of assessing the respective contributions of physical activity, sitting behavior, training timing, chronotype, and other lifestyle factors to sleep quality.

## 5. Conclusions

This exploratory study showed that amateur soccer players and physically active individuals reported better sleep quality than individuals with high sitting time, whereas no significant between-group differences were observed for Mediterranean diet adherence or chronotype. The similar sleep profiles observed in soccer players and physically active individuals do not support a soccer-specific advantage. Rather, better sleep quality was observed in both groups characterized by regular sport or structured physical activity compared with individuals characterized by high sitting time. However, given the cross-sectional design and the broader behavioral and lifestyle differences between groups, the independent contributions of physical activity, sitting behavior, and other potentially relevant factors cannot be determined.

The exploratory association between chronotype and sleep quality emerged only among individuals with high sitting time, with poorer sleep quality tending to co-occur with greater evening preference. However, this finding should be considered exploratory and hypothesis-generating. Therefore, this association provides a basis for future studies with larger sample sizes and longitudinal designs that use objective assessments of physical activity, sitting behavior, sleep, and relevant lifestyle factors to clarify the relationships underlying these findings.

## Figures and Tables

**Table 1:** Participant characteristics, expressed as mean ± standard deviation (median).

Variable	Soccer Players	Physically Active Individuals	Individuals with High Sitting Time
	*n* = 20	*n* = 18	*n* = 17
Age (years)	23.40 ± 4.16 (22.50)	22.78 ± 2.82 (23.00)	23.94 ± 1.71 (24.00)
Body mass (kg)	75.85 ± 12.96 (74.00)	74.61 ± 9.18 (75.00)	79.59 ± 14.40 (72.00)
Body height (cm)	178.95 ± 6.91 (180.00)	175.44 ± 4.96 (174.50)	178.00 ± 9.75 (177.00)
BMI (kg/m^2^)	23.58 ± 2.81 (23.35)	24.18 ± 2.30 (24.51)	25.00 ± 3.36 (24.31)
Sitting time (h/day)	NA	2.55 ± 0.84 (2.75)	6.96 ± 2.13 (6.70)

*n* = number; BMI = body mass index; NA = not assessed.

**Table 2:** Means and standard deviations of PSQI, PREDIMED, and MEQ scores across groups.

Variable	Soccer Players	Physically Active Individuals	Individuals with High Sitting Time
PSQI score	3.80 ± 1.32 *	4.17 ± 1.92 *	6.24 ± 2.05
PREDIMED score	6.80 ± 2.22	7.50 ± 1.89	7.29 ± 2.42
MEQ score	51.60 ± 5.90	50.89 ± 7.70	51.82 ± 11.43

PSQI = Pittsburgh Sleep Quality Index; PREDIMED = PREvención con DIeta MEDiterránea; MEQ = Morningness–Eveningness Questionnaire. * Significantly different (*p*-value < 0.05) when compared with individuals with high sitting time.

**Table 3:** Exploratory Pearson correlations of PSQI scores with PREDIMED and MEQ scores across groups.

Correlation	Soccer Players		Physically Active Individuals		Individuals with High Sitting Time	
	*r *(95% CI)	*p*	*r *(95% CI)	*p*	*r *(95% CI)	*p*
PSQI-PREDIMED	−0.014 (−0.454 to 0.431)	0.952	0.415 (−0.065 to 0.739)	0.087	–0.166 (−0.599 to 0.342)	0.523
PSQI-MEQ	–0.112 (−0.528 to 0.348)	0.638	–0.210 (−0.616 to 0.285)	0.403	−0.514 (−0.797 to −0.044)	0.035

PSQI = Pittsburgh Sleep Quality Index; PREDIMED = PREvención con DIeta MEDiterránea; MEQ = Morningness–Eveningness Questionnaire; *r* = Pearson’s correlation coefficient; CI = confidence intervals; *p* = *p*-value.

## Data Availability

Data are available in a publicly accessible repository. The original data presented in the study are openly available in GitHub (GitHub Inc., San Francisco, CA, USA) at: https://github.com/ccortis/DataSleep.git (accessed on 13 December 2025).

## Abbreviations

The following abbreviations are used in this manuscript:

IPAQ	International Physical Activity Questionnaire
HEPA	Health-enhancing physical activity
BMI	Body mass index
NA	Not assessed
PSQI	Pittsburgh Sleep Quality Index
PREDIMED	Prevención con Dieta Mediterránea
MEQ	Morningness–Eveningness Questionnaire
%	Percentage
ANOVA	Analysis of variance
η^2^	Eta squared
CI	Confidence interval
*p*	*p*-value
SE	Standard error
χ^2^	Chi-square
*r*	Pearson’s correlation coefficient
